# Anti-Biofilm Activity of Cell-Free Supernatant of *Saccharomyces cerevisiae* against *Staphylococcus aureus*

**DOI:** 10.4014/jmb.2008.08053

**Published:** 2020-09-21

**Authors:** Yeon Jin Kim, Hwan Hee Yu, Yeong Jin Park, Na-Kyoung Lee,, Hyun-Dong Paik

**Affiliations:** Department of Food Science and Biotechnology of Animal Resources, Konkuk University, Seoul 05029, Republic of Korea

**Keywords:** Anti-biofilm, *Saccharomyces cerevisiae*, cell-free supernatant, grapefruit seed extract, *Staphylococcus aureus*

## Abstract

*Staphylococcus aureus* is one of the most common microorganisms and causes foodborne diseases. In particular, biofilm-forming *S. aureus* is more resistant to antimicrobial agents and sanitizing treatments than planktonic cells. Therefore, this study aimed to investigate the anti-biofilm effects of cell-free supernatant (CFS) of *Saccharomyces cerevisiae* isolated from cucumber *jangajji* compared to grapefruit seed extract (GSE). CFS and GSE inhibited and degraded *S. aureus* biofilms. The adhesion ability, auto-aggregation, and exopolysaccharide production of CFS-treated *S. aureus*, compared to those of the control, were significantly decreased. Moreover, biofilm-related gene expression was altered upon CFS treatment. Scanning electron microscopy images confirmed that CFS exerted anti-biofilm effects against *S. aureus*. Therefore, these results suggest that *S. cerevisiae* CFS has anti-biofilm potential against *S. aureus* strains.

## Introduction

Foodborne diseases caused by pathogens pose a great threat to human health. According to the Center for Disease Control and Prevention (CDC), foodborne diseases are caused by *Salmonella* spp., *Clostridium perfringens*, *Campylobacter* spp., and *Staphylococcus aureus*, the most common microorganisms found in food eaten in the United States [1].

Most foodborne pathogens can form biofilms on various surfaces, such as stainless steel, glass, polyurethane, and rubber [2,3]. Biofilms formed by foodborne pathogens have become a major problem in meat, dairy, and fish processing, as well as in the ready-to-eat (RTE) food industry. Biofilm formation is an important factor in the cross-contamination and persistence of foodborne pathogens [4]. It is difficult to remove foodborne pathogens once they form biofilms because biofilm cells are more resistant to antimicrobial agents and sanitizing treatments than planktonic cells [5]. Biofilms are surrounded by extracellular polymeric substances, which are composed of exopolysaccharides (EPS), proteins, lipids, and extracellular DNA [6]. Extracellular polymeric substances protect bacterial cells from the effects of antimicrobial agents and assist in retaining the structural integrity of the biofilm.

*S. aureus* is a major foodborne pathogen that causes 241,148 illnesses per year [7] and forms biofilms on food and food contact surfaces [8]. Moreover, biofilm formation by *S. aureus* plays a crucial role in staphylococcal infections by protecting the colony from the host immune system and antimicrobial treatment [9]. Therefore, the prevention of *S. aureus* biofilm formation has become important for countering foodborne staphylococcal outbreaks.

Since antibiotics are limited in their ability to inhibit *S. aureus* biofilms, many studies on natural antimicrobial agents and compounds produced using probiotics have been performed [10-13]. For example, some natural preservatives, such as grapefruit seed extract (GSE), have been used together with antimicrobial and anti-biofilm agents against Gram-positive and Gram-negative bacteria [14]. Moreover, some probiotics consisting of lactic acid bacteria have been reported to inhibit biofilm formation by *Listeria monocytogenes* [4], *Pseudomonas aeruginosa* [15], *Bacillus licheniformis* [16], *Vibrio* spp. [17], and methicillin-resistant *S. aureus* (MRSA) [18].

*Saccharomyces cerevisiae* has been reported that the probiotic yeast isolated from fermented food such as kefir, wine, and cucumber *jangajji* (Korean fermented food) [19,20]. The positive effects of *S. cerevisiae*, including antioxidant, anti-inflammatory, toxin eradication, and antagonistic activities, have been investigated [21-23]. In particular, the supernatant and lysate of *S. cerevisiae* cultures exerted an anti-biofilm effect against *S. aureus*, as demonstrated by the expression of the α-hemolysin and enterotoxin A genes (*hla* and *sea*). However, the effects of *S. cerevisiae* on the cell surface characteristics, EPS production, and expression of biofilm-related genes in *S. aureus* have not yet been investigated.

Therefore, the present study aimed to investigate the anti-biofilm effects of cell-free supernatant (CFS) of *S. cerevisiae* isolated from cucumber *jangajji* against *S. aureus*. The anti-biofilm mechanisms were evaluated by investigating the differences in cell surface characteristics (adhesion ability, auto-aggregation, and hydrophobicity), EPS production, and biofilm-related gene expression compared to treatment with GSE. Moreover, *S. aureus* biofilms formed on glass coupons were observed by scanning electron microscopy (SEM).

## Materials and Methods

### Strains and Growth Conditions

Five strains of *S. cerevisiae* (KU200270, KU200278, KU200280, KU200281, and KU200284) isolated from cucumber *jangajji* were used in this study [19,20]. Nine strains of *S. aureus* (P130, P131, P86, P87, P88, P89, ATCC 6538, ATCC 12692, and ATCC 25923) were screened to investigate their biofilm-forming capacity. *S. cerevisiae* strains were cultured in yeast mold (YM) broth (Difco Laboratories, USA) at 25°C for 48 h and *S. aureus* was cultured in tryptic soy broth (TSB; Difco Laboratories) at 37°C for 24 h. All strains were stored at -80°C in a 20%glycerol solution.

### CFS Preparation

*S. cerevisiae* strains were inoculated in YM broth and cultures were agitated at 150 rpm at 25°C for 48 h. After incubation, *S. cerevisiae* culture medium was centrifuged at 15,420 ×*g* at 4°C for 15 min and the pH was adjusted to 6.5 ± 0.3 using 1 M NaOH. Then, the supernatant was filtered through a syringe filter (0.45-μm pore size)(Advantec, Japan) and stored at -80°C.

### Evaluation of Anti-Biofilm Effect by Minimum Inhibitory Concentration (MIC)

To investigate the MIC of GSE (ES Food, Korea), the double broth dilution method was used [14]. CFS and GSE were dissolved in YM broth and YM broth with 0.1% Tween 80, respectively, and serially diluted two-fold. *S. aureus* were cultured in TSB at 37°C for 24 h and then diluted in TSB to obtain a final concentration of 105 colony-forming units (CFU)/ml. Bacterial cultures (50 μl) in TSB supplemented with 50 μl of CFS or GSE in YM broth were transferred to 96-well polystyrene plates (SPL, Korea), which were incubated at 37°C for 24 h. After incubation, the lowest concentrations of CFS and GSE that could inhibit visible *S. aureus* growth were defined as MICs.

### Biofilm Inhibition Assay and Biofilm Degradation Assay

Biofilm inhibition and degradation by CFS were investigated by crystal violet (CV) assay [24]. To this purpose, the strains were treated with CFS and GSE at 1/2 × MIC based on the MIC related to each strains.

To examine the biofilm inhibition effects of CFS and GSE, bacterial suspension (50 μl) treated with CFS or GSE (50 μl) were transferred to a 96-well polystyrene plate and incubated at 37°C for 24 h; the control was treated with YM broth. Following incubation, cell suspensions were removed, and the wells were washed twice with 150 μl of distilled water (DW). The biofilm cells were dried at 37°C for 20 min and then stained using 1% CV solution (150 μl) for 30 min. The CV solution was removed, and the plate was washed twice with cold water. The biofilm cells were treated with dissolving solution (150 μl; 30% methanol and 10% acetic acid) to measure the optical density (OD) of the CV solution at 570 nm using a microplate reader (Molecular Devices, USA).

To examine the biofilm degradation effects of CFS and GSE, bacterial suspensions (100 μl) were transferred to a 96-well polystyrene plate and incubated at 37°C for 24 h. Following incubation, the bacterial suspensions were removed and treated with CFS or GSE (100 μl). The control was treated with YM broth. After incubation at 37°C for 24 h, biofilm quantification was performed as described above.

The biofilm inhibition and degradation rate (%) was calculated using the following equation:

Biofilm inhibition and degradation rate (%) = (1-OD_treatment_/OD_control_) × 100

### Adhesion Ability to the Glass Surface

The adhesion ability to *S. aureus* strains to the glass surface was determined according to Islam *et al*. [25] with some modifications. Briefly, bacterial cultures (5 ml) diluted in TSB to obtain a final concentration of 10^6^ CFU/ml were transferred to a glass tube and mixed with CFS or GSE (5 ml) at 1/2 × MIC; the control was treated with YM broth. Then, the glass tubes were placed at an angle of 30° and incubated at 37°C for 24 h. Following incubation, the planktonic cells was removed, and the tube was washed with phosphate buffered solution (PBS, pH 7.4; Hyclone, USA). The adhered cells were washed with PBS and re-suspended in fresh PBS (10 ml). The absorbance of the adhered cells was measured at 600 nm (OD_adhered cell_). Then, the adhered cells were mixed with the planktonic cells and washed with PBS, and the absorbance of the cell mixture was measured at 600 nm (OD_cell mixture_). The adhesion ability (%) was calculated using the following equation:

Adhesion ability (%) = (OD_adhered cell_/OD_cell mixture_) × 100

### Auto-Aggregation Ability Assay

The auto-aggregation ability of *S. aureus* was assayed according to the methods described by Song *et al*. [14] with some modifications. Briefly, bacterial cells treated with CFS or GSE at 1/2 × MIC, or YM broth were incubated at 37°C for 24 h. Following incubation, the cell suspensions were centrifuged at 17,709 ×*g* at 4°C for 5 min and washed twice with PBS. The washed cells were resuspended in fresh PBS, and the OD_600_ was adjusted to 0.5 ± 0.05 (OD_initial_). Absorbance-adjusted cell suspensions (4 ml) were incubated at 37°C for 8 h. After incubation, the absorbance of the upper layer was measured at 600 nm (OD_treated_). Auto-aggregation (%) was calculated using the following equation:

Auto-aggregation (%) = (1- OD_treated_ /OD_initial_) × 100

### Hydrophobicity Assay

Hydrophobicity was investigated according to a previously described protocol [24]. Absorbance-adjusted cells were prepared as described above (OD_initial_). Then, cell suspensions (2 ml) were mixed with chloroform (0.5 ml), and the tubes were vortexed for 2 min. Following incubation for 15 min, the absorbance of the aqueous layer was measured at 600 nm (OD_treated_). Hydrophobicity (%) was calculated using the following equation:

Hydrophobicity (%) = (1- OD_treated_/OD_initial_) × 100

### EPS Production

To examine EPS production, the phenol-sulfuric acid method was performed as described by Chiba *et al*. [26]. Bacterial cultures diluted in TSB to obtain a final concentration of 10^7^ CFU/ml were mixed with CFS or GSE (5 ml) at 1/2 × MIC and incubated at 37°C for 24 h. The control was treated with YM broth. After incubation, cell suspensions were centrifuged at 8,000 ×*g* at 25°C for 10 min and then 1.5 M NaCl (1 ml) was added. Then, the cell suspension was re-centrifugated at 5,000 ×*g* at 25°C for 10 min, and the supernatants (60 μl) were mixed with 5%phenol (60 μl) and sulfuric acid (4 ml). After incubation at 30°C for 10 min, OD_490_ was measured. EPS production (%) was calculated using the following equation:

EPS quantification (%) = (OD_treatment_/OD_control_) × 100

### Biofilm-Related Gene Expression

*S. aureus* ATCC 12692 was cultured in TSB at 37°C for 24 h, and the bacterial cultures were diluted in TSB to obtain a final concentration of 10^6^ CFU/ml. The diluted cell suspensions were treated with CFS of *S. cerevisiae* KU200278 or GSE at 1/2 × MIC; the control was treated with YM broth. After treatment, total RNA was extracted using TRIzol (Invitrogen, Thermo Fisher Scientific, USA) and complementary DNA (cDNA) was synthesized using the cDNA synthesis kit. Then, reverse transcription-polymerase chain reaction real-time PCR (RT-PCR) was performed to evaluate the expression of biofilm-related genes using specific primers (Table 2). The results were calculated using the ΔΔCt method.

### SEM Analysis

To investigate morphological changes in treated bacterial cells, SEM analysis of *S. aureus* ATCC 12692 treated with CFS of *S. cerevisiae* KU200278 or GSE at 1/2 × MIC on a glass coupon was performed. Prior to microscopic analysis, *S. aureus* ATCC 12692 was cultured in TSB at 37°C for 24 h, and then the grown bacterial culture was diluted in TSB to obtain a final concentration of 10^6^ CFU/ml. To estimate the biofilm inhibition effects of CFS and GSE, bacterial cultures (2 ml) were treated with CFS or GSE on glass coupons. The biofilm degradation effects of CFS and GSE were determined on glass coupons using pre-formed biofilms treated with CFS or GSE. The control was treated with YM broth. Following treatment, planktonic cells were washed twice with PBS, and biofilm cells were fixed with 2.5% glutaraldehyde in PBS at 4°C for 1 h. Then, the fixed samples were washed twice with PBS and dehydrated using ethanol at graded concentrations (50%, 70%, 80%, 90%, and 100%) for 15 min (for each concentration). After dehydration, ethanol was replaced with isoamyl acetate, and the samples were freeze-dried for 48 h. To observe the biofilm structure, the samples were coated with gold (15 mV for 1.5 min) and observed using a field emission scanning electron microscope (FESEM; SU8010; Hitachi High-Technologies Co., Japan).

### Statistical Analysis

All experiments were performed in triplicate. Statistical analysis was performed by SPSS version 18.0 (SPSS Inc., USA). The results were presented as the mean ± standard error. Significant differences among the mean values were evaluated by one-way analysis of variance (ANOVA).

## Results and Discussion

### MIC of CFS for *S. aureus*

Among the nine *S. aureus* strains, three strains (ATCC 6538, ATCC 12692, and ATCC 25923) showed high biofilm-forming capacity (data not shown) and were hence used for further study. The antimicrobial effects of CFS and GSE against *S. aureus* were confirmed; however, CFS did not inhibit the growth of *S. aureus*. On the other hand, the MIC of GSE that exerts antimicrobial effects against *S. aureus* ATCC 6538, ATCC 12692, and ATCC 25923 was 100, 50, and 200 μg/ml, respectively (Table 1). According to Heggers *et al*. [27], GSE damages the cell membrane, degrades cytoplasmic components, and finally induces the cell death. *S. cerevisiae* was reported on anti-biofilm effects by diverse enzyme, peptides, and antibiotics [12]. Conversely, the anti-biofilm effects of *S. cerevisiae* have been reported to depend on the activity of diverse enzymes, peptides, and antibiotics [12]. Therefore, *S. cerevisiae* CFS is supposed to show better anti-biofilm effects compared to GSE.

### Effect of CFS on Biofilm Inhibition and Degradation

**Fig. 1:**
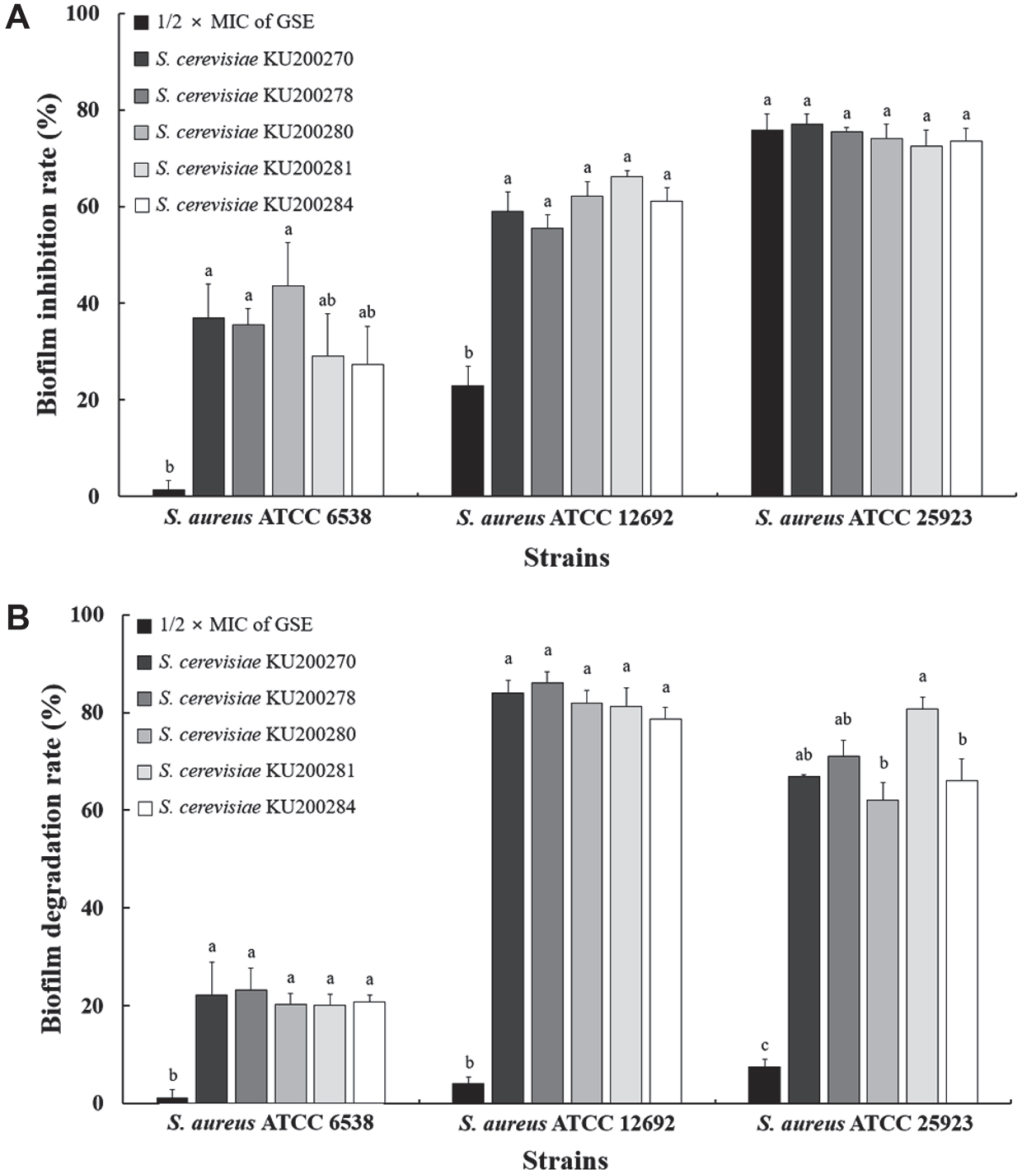
Effects of cell-free supernatant (CFS) of *S. cerevisiae* strains on *S. aureus* biofilms. (**A**) Biofilm inhibition effect of grapefruit seed extract (GSE) at 1/2 × minimum inhibitory concentration (MIC) and CFS; (**B**) biofilm degradation effect of GSE at 1/2 × MIC and CFS. The MIC of GSE against *S. aureus* ATCC 6538, *S. aureus* ATCC 12692, and *S. aureus* ATCC 25923 was 50, 25, and 100 μg/ml, respectively. All experiments were performed in triplicates, and reported values represent the mean ± standard error. ^a-c^Different letters in the same bar indicate significant differences (*p* < 0.05).

To compare the effect of CFS and GSE on bacteria, cells were treated with CFS or GSE at 1/2 × MIC [24,28]. The inhibitory effects of CFS and GSE on *S. aureus* biofilm are shown in Fig. 1A. CFS and GSE significantly inhibited biofilm formation by the three *S. aureus* strains (*p* < 0.05). Interestingly, the biofilm inhibition rates of CFS-treated *S. aureus* ATCC 6538 and ATCC 12692 ranged from 27.27% to 43.66% and from 55.55% to 66.29%, respectively. On the other hand, GSE treatment inhibited *S. aureus* ATCC 6538 and ATCC 12692 biofilm formation by 1.39%and 22.96%, respectively. No significant difference was observed between CFS- and GSE-dependent biofilm inhibition of *S. aureus* ATCC 25923 (*p* > 0.05). The effects of CFS and GSE on *S. aureus* biofilm degradation are shown in Fig. 1B. The mature *S. aureus* biofilms were significantly degraded by both CFS and GSE (*p* < 0.05). In particular, CFS degraded *S. aureus* biofilms at a rate ranging from 20.01% to 86.04%, whereas GSE degraded the biofilms at a rate ranging from 1.16% to 7.53%.

Similar to our results, previous reports showed that the supernatant and lysate of *S. cerevisiae* cultures inhibited *S. aureus* and *P. aeruginosa* biofilm formation [12, 29]. Moreover, the supernatant and lysate of *S. cerevisiae* exerted anti-biofilm effects against *S. aureus*, inhibiting biofilm formation of methicillin-sensitive *S. aureus* (MSSA) and MRSA strains by 48% and 69%, respectively. Mannoproteins, components of the *S. cerevisiae* cell wall, also inhibited biofilm formation and dispersion. Moreover, the anti-biofilm effects of GSE against *S. aureus* and Escherichia coli were also investigated [14]. Notably, 1/2 × MIC GSE-treated *S. aureus* and *E. coli* displayed inhibited biofilm formation and maturation. In particular, GSE at 1/2 × MIC showed inhibition rates of *S. aureus* and *E. coli* biofilm of 44.2% and 29.8%, respectively, and biofilm degradation rates of 35.2% and 36.9%, respectively.

### Cell Surface Characteristics and EPS Production of CFS-treated *S. aureus*

To investigate the anti-biofilm mechanisms of CFS, the adhesion ability, auto-aggregation ability, hydrophobicity, and EPS production of *S. aureus* were examined. Table 3 shows the characteristics of *S. aureus* strains treated with CFS of *S. cerevisiae* KU200278, which showed the most effective anti-biofilm potential among CFSs from the yeast strains or 1/2 × MIC GSE. Adhesion, auto-aggregation, hydrophobicity, and EPS production are related to bacterial attachment and biofilm formation rate [30, 31].

CFS treatment of all tested strains and GSE treatment of *S. aureus* ATCC 25923, compared with the control treatment (85.28%-94.32%), significantly decreased the adhesion ability to the glass surface (48.42%-72.95%)(*p* < 0.05). However, GSE treatment of *S. aureus* ATCC 6538 and ATCC 12692 significantly increased the adhesion ability (*p* < 0.05). On the other hand, auto-aggregation and EPS production of *S. aureus* treated with CFS or GSE at 1/2 × MIC, compared to those of the control, significantly decreased (*p* < 0.05). Specifically, *S. aureus* strains treated with CFS showed significantly reduced auto-aggregation and EPS production with respect to strains treated with GSE at 1/2 × MIC (*p* < 0.05). However, the effects of treatments on *S. aureus* hydrophobicity differed depending on the strain. Nevertheless, CFS did not significantly affect the hydrophobicity of any of the tested strains (*p* > 0.05).

Biofilm formation is followed by adhesion to the surface, auto-aggregation, attachment via hydrophobic interactions, and EPS production [3, 32, 33]. Therefore, CFS and GSE affected all steps of biofilm formation: 1) the initial step of adhesion, attachment, and biofilm formation of *S. aureus*; 2) the step of attachment by auto-aggregation and establishment of hydrophobic interactions; and 3) the step of maturation by EPS production.

### Effect of CFS on Expression of Biofilm-Related Gene

The effects of CFS on the expression of biofilm-related genes of *S. aureus* were determined by qRT-PCR. Polysaccharide intercellular adhesin (PIA), a component of the polysaccharide slime of *S. aureus*, is synthesized by the intercellular adhesion (*ica*) operon and plays an important role in biofilm formation and adhesion [34]. The *ica* operon consists of the *icaABCD* cluster and *icaR*; among operon components, *icaA* is the essential regulator of *icaD* expression, which mediates PIA biosynthesis [32].

**Fig. 2:**
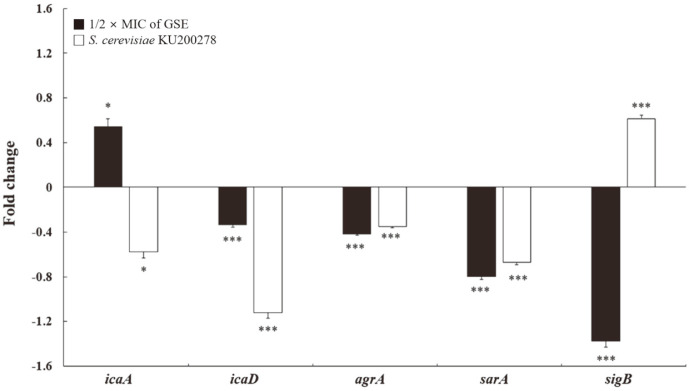
Effects of cell-free supernatant (CFS) of *S. cerevisiae* KU200278 on gene expression of *S. aureus* ATCC 12692. All experiments were performed in triplicates, and reported values represent the mean ± standard error (*p < 0.05, **p < 0.01, ***p < 0.001). Grapefruit seed extract (GSE) at 1/2 × minimum inhibitory concentration (MIC); *icaA* and *icaD*, intercellular adhesion; *agrA*, accessory gene regulator; *sarA*, Staphylococcal accessory regulator; *sigB*, modulator of the expression of the virulence gene *agrA* and *sarA*.

As shown in Fig. 2, CFS and GSE at 1/2 × MIC altered the expression of *ica* operon genes in *S. aureus*. Indeed, the expression of *icaA* and *icaD* in CFS-treated *S. aureus* was significantly downregulated (*p* < 0.05), whereas the expression of *icaA* in GSE-treated *S. aureus* was significantly upregulated (*p* < 0.05). However, the expression of *icaR* in *S. aureus* treated with GSE or CFS did not significantly change compared with the control (data not shown). Therefore, CFS altered *icaA* and *icaD* expression by decreasing biofilm formation and adhesion ability.

The virulence genes accessory gene regulator (*agrA*) and staphylococcal accessory regulator (*sarA*) also affect biofilm formation [8]. In fact, *agrA* and *sarA* regulate the quorum sensing system and the secretion of adhesion factors, thereby inhibiting initial adhesion and cell-cell interactions, respectively [31,32]. Notably, *sigB* modulates the expression of *agrA* and *sarA* during stress response [32].

Fig. 2 shows that CFS or GSE treatment triggered the downregulation of *agrA* and *sarA* expression in *S. aureus*. In particular, *agrA* expression was significantly downregulated by 0.35-fold and 0.42-fold upon CFS and GSE treatment, respectively. Moreover, *sarA* expression was significantly downregulated by 0.67-fold and 0.79-fold upon CFS and GSE treatment, respectively. However, *sigB* expression was upregulated by 0.61-fold upon CFS treatment, while being downregulated by 1.37-fold upon GSE treatment. Therefore, *agrA* and *sarA* downregulation and *sigB* upregulation could affect the inhibition and degradation of *S. aureus* biofilm.

### SEM Analysis of *S. aureus* ATCC 12692 on Glass Surface

**Fig. 3:**
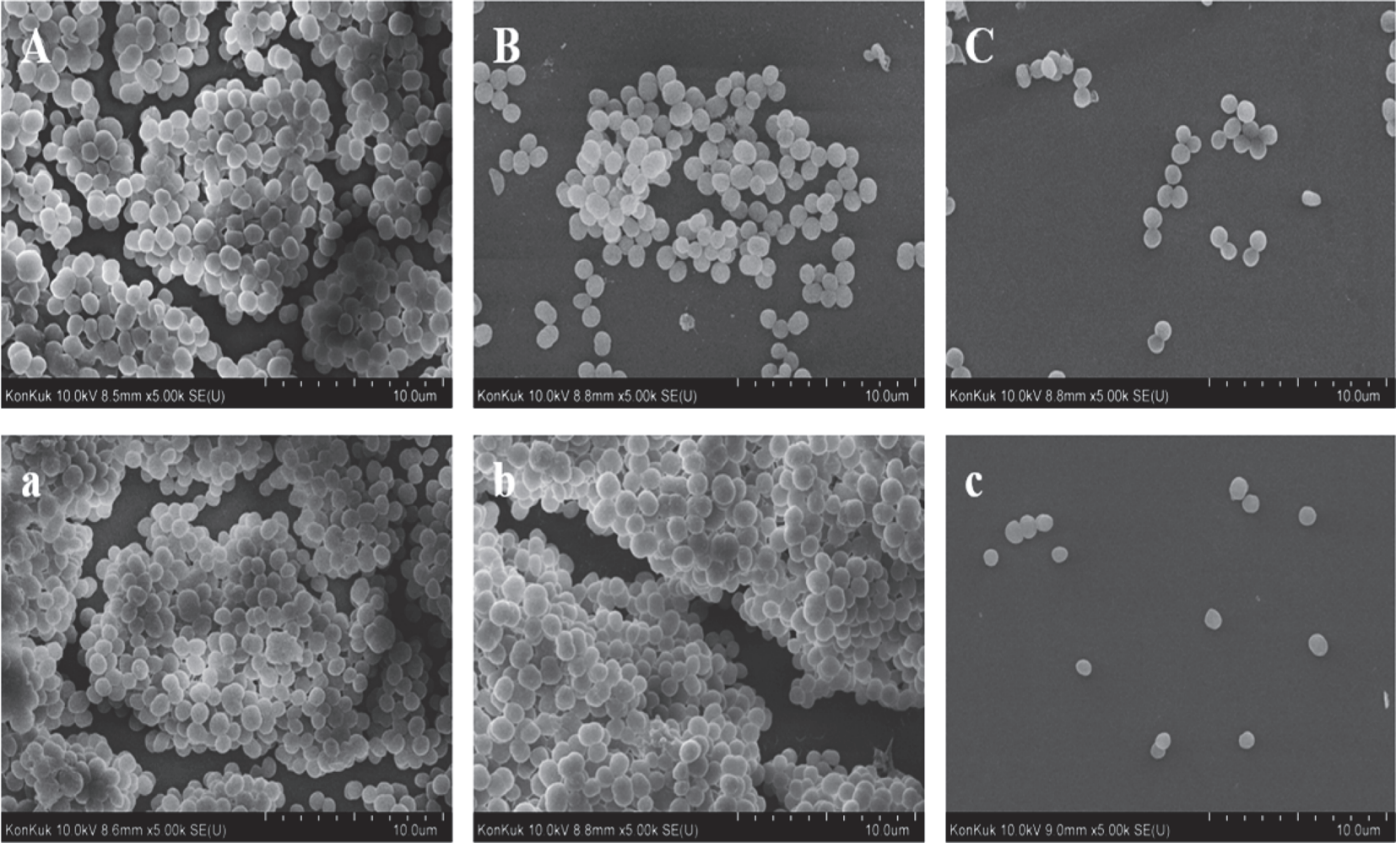
Scanning electron microscopy analysis of *S. aureus* ATCC 12692 treated with cell-free supernatant (CFS) of *S. cerevisiae* KU200278 on glass coupons (× 10,000 magnification). (**A**-**C**) Biofilm inhibition; (**A**) control; (**B**) treatment with grapefruit seed extract (GSE) at 1/2 × minimum inhibitory concentration (MIC); (**C**) CFS treatment; (**a**-**c**), biofilm degradation; (**a**) control; (**b**) treatment with GSE at 1/2 × MIC; (**c**) CFS treatment.

To confirm the morphological changes of *S. aureus* ATCC 12692 treated with CFS of *S. cerevisiae* KU200278, SEM analysis was performed (Fig. 3). The biofilm inhibition and degradation effects of CFS and GSE were also investigated. In the control *S. aureus* groups, complex and clumped biofilm structures with thick and multiple layers were formed (Figs. 3A and 3a). On the other hand, CSF-treated biofilms were single layered and showed reduced attachment of *S. aureus* (Figs. 3B and 3b). Moreover, the images of *S. aureus* treated with GSE at 1/2 × MIC were well correlated with the measured anti-biofilm effects. Indeed, GSE inhibited biofilm formation by decreasing cell attachment and aggregation (Fig. 3C). Nevertheless, GSE treatment, unlike CFS treatment, did not trigger *S. aureus* biofilm degradation (Fig. 3c). In fact, GSE-treated mature biofilms showed clumped, complex, and multi-layered structures. These results indicated that CFS from *S. cerevisiae* KU200278 showed considerable anti-biofilm potential against *S. aureus*.

In conclusion, this study investigated the anti-biofilm effect of CFS of a *S. cerevisiae* isolate against *S. aureus*. CFS significantly inhibited biofilm formation and degraded mature biofilms of *S. aureus* strains. Notably, the adhesion ability, auto-aggregation ability, and EPS production of CFS-treated *S. aureus*, compared to those of the control, were significantly decreased. Furthermore, RT-PCR showed that CFS altered biofilm-related gene expression. Finally, SEM images confirmed that CFS inhibited and degraded *S. aureus* biofilms. Altogether, these results suggested that CFS of *S. cerevisiae* isolated from cucumber *jangajji* showed anti-biofilm potential against MSSA and MRSA strains.

## Figures and Tables

**Table 1:** Minimum inhibitory concentration (MIC) of grapefruit seed extract against *S. aureus*.

Strain	MIC (μg/ml)
*S. aureus* ATCC 6538	100
*S. aureus* ATCC 12692	50
*S. aureus* ATCC 25923	200

All experiments were performed in triplicates.

**Table 2:** Sequences of primers used for real-time PCR analysis.

Gene	Primers sequence (5’to 3’)	Reference
*icaA*	F: CTGGCGCAGTCAATACTATTTCGGGTGTCT	[31]
	R: GACCTCCCAATGTTTCTGGAACCAACATCC	
*icaD*	F: ACCCAACGCTAAAATCATCG	[30]
	R: GCGAAAATGCCCATAGTTTC	
*icaR*	F: CAATAATCTAATACGCCTGAG	[28]
	R: AGTAGCGAATACACTTCATCT	
*agrA*	F: TGATAATCCTTATGAGGT GCTT	[31]
	R: CACTGTGACTCGTAACGAAAA	
*sarA*	F: CAAACAACCACAAGTTGTTAAAGC	[31]
	R: TGTTTGCTTCAGTGATTCGTTT	
*sigB*	F: AGTGTTAGAAGCAATGGAAATG	[32]
	R: CGATACGCTCACCTGTCTCT	
16S *rRNA*	F: ACTCCTACGGGAGGCAGCAG	[31]
	R: ATTACCGCGGCTGCTGG	

**Table 3:** Effects of cell-free supernatant (CFS) of *S. cerevisiae* KU200278 on cell surface characteristics (adhesion ability, auto-aggregation ability, and hydrophobicity) and exopolysaccharide (EPS) production of *S. aureus*.

Treatment	Control	GSE at 1/2 × MIC	CFS
Adhesion ability (%)			
*S. aureus* ATCC 6538	88.79±0.30^b^	89.86±0.80^c^	58.29±0.66^a^
*S. aureus* ATCC 12692	94.32±0.97^b^	98.38±0.48^c^	64.02±0.16^a^
*S. aureus* ATCC 25923	85.28±0.00^c^	48.42±0.56^a^	72.95±0.53^b^
Auto-aggregation (%)			
*S. aureus* ATCC 6538	51.03±0.13^c^	47.52±0.48^b^	32.10±0.76^a^
*S. aureus* ATCC 12692	73.54±0.36^c^	69.15±0.31^b^	53.52±0.56^a^
*S. aureus* ATCC 25923	65.05±0.57^c^	60.57±0.55^b^	31.56±0.49^a^
Hydrophobicity (%)			
*S. aureus* ATCC 6538	92.36±0.00^a^	93.14±0.24^b^	92.97±0.06^ab^
*S. aureus* ATCC 12692	94.03±0.00^a^	94.11±0.17^a^	94.08±0.13^a^
*S. aureus* ATCC 25923	87.04±0.16^a^	89.73±0.16^b^	87.46±0.14^a^
EPS quantification (%)			
*S. aureus* ATCC 6538	100±0.45^c^	41.96±0.45^b^	35.27±1.18^a^
*S. aureus* ATCC 12692	100±1.00^c^	58.00±0.50^b^	26.50±0.50^a^
*S. aureus* ATCC 25923	100±0.55^c^	63.44±1.16^b^	41.22±0.82^a^

1/2 × minimum inhibitory concentration (MIC) of grapefruit seed extract (GSE) (*S. aureus* ATCC 6538, 50 μg/ml; *S. aureus* ATCC 12692, 25 μg/ml; *S. aureus* ATCC 25923, 100 μg/ml).

All experiments were performed in triplicates, and reported values represent the mean ± standard error.

^a-c^Values with different letters in the same column are significantly different (*p* < 0.05).
